# For 19-Month-Olds, What Happens On-Screen Stays On-Screen

**DOI:** 10.1162/opmi_a_00043

**Published:** 2021-09-10

**Authors:** Barbu Revencu, Gergely Csibra

**Affiliations:** Cognitive Development Center, Department of Cognitive Science, Central European University; Cognitive Development Center, Department of Cognitive Science, Central European University; Birkbeck College, University of London

**Keywords:** cognitive development, representations, animation, fiction, methodology

## Abstract

Humans rely extensively on external representations such as drawings, maps, and animations. While animations are widely used in infancy research, little is known about how infants interpret them. In this study, we asked whether 19-month-olds take what they see on a screen to be happening here and now, or whether they think that on-screen events are decoupled from the immediate environment. In Experiments 1–3, we found that infants did not expect a falling animated ball to end up in boxes below the screen, even though they could track the ball (i) when the ball was real or (ii) when the boxes were also part of the animation. In Experiment 4, we tested whether infants think of screens as spatially bounded physical containers that do not allow objects to pass through. When two location cues were pitted against each other, infants individuated the protagonist of an animation by its virtual location (the animation to which it belonged), not by its physical location (the screen on which the animation was presented). Thus, 19-month-olds reject animation-reality crossovers but accept the depiction of the same animated environment on multiple screens. These results are consistent with the possibility that 19-month-olds interpret animations as external representations.

## INTRODUCTION

Humans rely extensively on *external representations* in communication: drawings for objects, maps for space, calendars for time, and language for virtually anything they can think of. This capacity allows humans to transcend their immediate environment and gather information about distal states of affairs from proximal sources by decoupling incoming percepts, which necessarily reach our senses *here* and *now* (e.g., an utterance spoken *at present*, a piece of paper *in front of me*), from the information carried by those percepts (Ittelson, 1996; Millikan, 2017).

Representations can carry information about at least two types of content. On the one hand, we use representations to convey information about *particular individuals* in the world: the proper name *Barack Obama* refers to the former president of the United States; a map of London represents the spatial layout of the same city; a child’s drawing of her teddy bear stands in for her favorite toy (while the toy itself does not represent any particular bear). On the other, the very same representational vehicles can be used to communicate about nonspecific or fictional entities too: the proper name *Batman* picks out a well-known fictional character; a map of Hogwarts represents spatial relations of a place we can never visit; a child’s drawing of a house need not pick out any particular house outside her mind. By definition, these entities can be accessed via representations only.

Even though both types of content have been investigated for linguistic representations and their development (e.g., Carlson, 1977; Chierchia, 1998; Gelman, 2003), many philosophers and psychologists working on visual representations have chosen to focus on those representations that stand in a one-to-one relation with things in the world (DeLoache, 1987, 1991, 2004; Fodor & Pylyshyn, 2015; Frege, 1975/1892; Goodman, 1976; Rakoczy et al., 2005; Recanati, 2012; Tomasello et al., 1999). Empirical research along these lines revealed that 2-year-olds are at chance in object retrieval tasks if they are shown where the object is via pictures, video, or scale models (DeLoache, 1991; Troseth & DeLoache, 1998). In these studies, children have to find a toy hidden by the experimenter in a room with which they had previously been familiarized. In picture studies, the experimenter shows children a photograph of the room, points to the corresponding location (e.g., to the chair), and tells them that she hid the toy there (DeLoache, 1991). In scale model studies, the experimenter does the same on a miniature version of the room (DeLoache, 1987). In video studies, children watch the entire hiding event on TV (Troseth & DeLoache, 1998). Since it is not before their third birthday that children reliably pass all three tasks, it has been concluded that grasping and exploiting external representations undergoes a protracted development because children have to overcome a *dual representation* problem (see DeLoache, 2004, for a review): a nonlinguistic representation represents an object while being an object itself.

Note, however, that all of these studies require children to link the information they obtain by means of representations to a particular state of affairs. However, as we have pointed out above, this is only part of the story, as there are representations that do not point to anything in the world. Thus, if representations of particular objects are only a subclass of external representations, the conclusions obtained from research on the subclass cannot be generalized to the entire class. After all, infants can perceptually discriminate between 2D representations and 3D objects before their first birthday (DeLoache et al., 1979), as well as identify the objects represented correctly. When treating photographs or videos as 3D objects (e.g., DeLoache et al., 1998; Pierroutsakos & Troseth, 2003), 9-month-olds adjust their grabbing behaviors to the depictions of objects whose life-size would afford picking up; in other words, they are less likely to pick up a picture of a bed than that of a bottle (DeLoache & Burns, 1994). Six months later, infants extend what they have learned from a representation to the depicted object kind. When a novel label (e.g., “ziff”) is paired with a drawing of a garbage disposal crusher, 15- and 18-month-olds correctly infer that the label applies to actual crushers as well (Geraghty et al., 2014; Preissler & Carey, 2004). It thus remains possible that understanding representations of particular objects and events may require the additional step of linking the information carried by visual representations to actual objects and events. Thus, whether and how nonlinguistic representations are interpreted and understood in development as a broad stimulus category is very much still an open question.

Consider, for instance, Heider and Simmel’s (1944) short animations of geometrical shapes moving around. When adults are asked to describe such clips, they respond as if they talked about real agents, attributing to them goals, desires, and intentions: the big triangle is chasing the small triangle, the circle wants to exit the enclosing, and the three shapes together form a love triangle (Heider & Simmel, 1944; Oatley & Yuill, 1985). Regardless, they are not fooled into believing that these shapes *do* form romantic bonds in front of them. Adults know these are not fully fledged agents: they are not afraid that the big bully triangle will chase them, and they do not consider interacting with the shapes. In other words, they know that the shapes and movement patterns *stand for* various agents and interactions among them even if they do not expect these events to have actually happened (absent additional information). We take the link between a spatiotemporally trackable object (e.g., a triangle) and a conceptually defined entity (e.g., an agent) to be constitutive of representational relations.

Animations inspired by Heider and Simmel’s minimalist stimuli are routinely used in developmental research to tap into the emergence of conceptual understanding and, in many cases, there is substantive evidence that young infants interpret them in an adult-like manner: they attribute instrumental and social goals to simple shapes (Gergely et al., 1995; Kuhlmeier et al., 2003; Liu et al., 2017), they infer social relations from minimal interactions between these shapes (Powell & Spelke, 2013; Tatone et al., 2015), as well as ascribe mental states to them (Surian et al., 2007; Tauzin & Gergely, 2018). Undoubtedly, infants’ inferences are prompted by the cues they would use to detect agents outside the lab, such as face-like features, self-propelled movement, and contingent responsivity (see Opfer & Gelman, 2011, for a review). But little is known about what infants make of these stimuli once the interpretive process has started.

Assuming that infants do not possess a concept of representation (Perner, 1991), how do they interpret animations? We delineate four hypotheses for infants’ interpretation of animations as a broad stimulus category. First, infants might find the animations *fully opaque* (Hypothesis 1) because the information contained therein is too sparse to be interpreted (i.e., they cannot see a circle as an agent, because agents are three-dimensional entities with whom one can interact contingently). At the opposite end, infants might be *naïve realists* with respect to animations and take animations to be spatiotemporally continuous with the surrounding environment (Hypothesis 2). If so, they should think that whatever is represented on a screen is happening here and now, in front of them. In-between the two extremes, infants might think that animations are temporally but not spatially continuous with the immediate environment. This would occur if infants have learned that screens have boundaries that cannot be crossed by objects and are perceiving screens as (spatially self-contained) *aquaria* (Hypothesis 3). Finally, it is possible that infants interpret animations as *representations*, though not necessarily of particular objects or states of affairs (Hypothesis 4). This would imply that infants (i) can establish a link between an object symbol (e.g., a coherent pixel constellation on the screen) and a spatiotemporally undefined referent (i.e., a fictional object); and (ii) dissociate between the two to the extent they have learned how the representational medium works (here, on-screen 2D animations).

**Figure 1.**
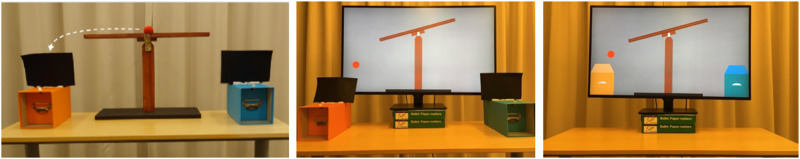
Setup overview in Experiments 1–3: Reality Baseline, Crossover, Animation (from left to right).

To test the first two hypotheses, *full opacity* and *naïve realism*, we investigated whether 19-month-olds expect a ball falling on the screen to land in boxes below the screen (Figure 1). First, we obtained a baseline for infants’ accuracy in tracking real balls falling in one of two boxes (Experiment 1, *Reality Baseline*). Second, we tested whether infants expect animated balls falling on the screen to land in boxes below the screen (Experiment 2, *Crossover*). Third, we ran a control version, in which both the ball and the boxes were part of the animation, to make sure that they can follow the animated ball’s trajectory when everything happens on the screen (Experiment 3, *Animation*). Finally, we tested whether infants think animations are tied to the screen on which they are presented (Experiment 4, *Aquarium*). We tested 19-month-olds because we sought for an age at which infants are known to fail DeLoache-type tasks but do not have problems understanding questions about objects’ locations. We have piloted Experiment 1 with 12- and 15-month-olds as well but could not get many of them to answer the experimenter’s questions. The hypotheses and methods for all experiments were preregistered at the Open Science Framework (Experiments 1 and 2: https://osf.io/bwu9p; Experiment 3: https://osf.io/juerf/; Experiment 4: https://osf.io/gj5ys/). The experiments were approved by United Ethical Review Committee for Research in Psychology (EPKEB) in Hungary, and informed consent was obtained from the participants’ caregivers before the experimental session.

## EXPERIMENT 1: REALITY BASELINE

### Methods

#### Participants

The final sample consisted of 16 typically developing 19-month-olds (*M*_age_ = 19 months 14 days, *SD*_age_ = 12.38 days). In the pilot ran for Experiment 1, 10 out of 10 babies answered the question on the first trial correctly. Based on this data, we ran a power analysis for the binomial test against chance with an assumed effect size of 0.875. This effect is detected with 85% power with a sample size of 15, but for counterbalancing reasons, we chose 16 as our sample size. The samples for the Experiments 2 and 3 were chosen based on this analysis in order to have equal samples across the three groups.

#### Apparatus and Materials

We built a wooden seesaw (height = 40 cm; width = 60 cm) that could be inclined left and right (angle ≈ 25°) by means of a 25-cm handle extending from the back of the seesaw, which allowed us to manipulate the seesaw from behind a curtain (Figure 1, left). We used several identical-looking red sponge balls (radius = 2.5 cm) and two different-colored rectangular cardboard boxes (14 × 15 × 26 cm^3^) as containers for the balls dropped from the seesaw. We initially thought that infants might want to open the boxes to retrieve the balls, which we sought to avoid because we did not want to give infants feedback on their choice. We therefore added a secret compartment to each box, which ensured that the balls in the box were not accessible to infants even if they tried to open the boxes. These compartments were padded with soft cloth to remove the acoustic cues produced by the falling ball. In addition, two black rectangular cardboards were attached on top of the boxes in Experiments 1 and 2 to cover the edge of the screen in Experiment 2. We used two plush toys (a cat and a bird), which were hidden in the boxes to familiarize infants with the task of pointing to object locations, and a canvas bag for storing the toys and balls throughout the procedure. Three ceiling-mounted video cameras recorded infants’ behavior from different angles.

#### Stimuli

A small loudspeaker, placed behind the seesaw, played a 1-s jingle before each test trial to prompt infants to attend to the ball-falling event. The experimenter talked to the participants using infant-directed intonation and following a prespecified script (see *Procedure*).

### Procedure

#### Familiarization

Infants were seated on their caregivers’ laps on a chair, approximately 40 centimeters from the table on which the seesaw was placed. The experimenter drew the infant’s attention to the two boxes, showed them that they can be opened, and revealed their (empty) insides. She then took a plush toy cat from a canvas bag and allowed the infant to inspect the toy for 10 s. Meanwhile, she pushed the inner compartments backwards, so she would be able to drop the toy into the boxes. She then asked the infant to hand the toy, moved behind the seesaw, drew the infant’s attention to herself (“[Infants’ name,] look!”), and dropped the toy into one of the two boxes. She then slid the inner compartments back into place, pushed the boxes to the edge of the table, where the infant could reach them, and asked “Where is it?” If the infant failed to respond within 3 s, she asked them “Where is the cat?” two more times (at 10-s intervals) before retrieving the toy from the box herself. If infants picked the right box, the experimenter congratulated the infant and took the toy out from the box. If infants picked the wrong one, the experimenter showed them that the box they chose was empty and retrieved the toy from the box where it had been dropped. The next familiarization trial was identical except that a toy bird replaced the cat and was dropped in the other box by the experimenter. When infants responded correctly for two trials in a row (out of a maximum of eight number of attempts), the experimenter put the toys away, pushed the boxes to the left and right of the seesaw, and pulled their inner compartments backwards so the ball could fall from the seesaw into the boxes.

#### Test

The test trials started with the experimenter drawing the infant’s attention to the ball that had been placed in the middle of the seesaw before the session. While looking at the ball from behind the seesaw, she drew the infant’s attention to the red ball in the middle of the seesaw (“[Name,] look at the ball!”). Immediately afterwards, infants heard a 1-s jingle coming from a loudspeaker behind the seesaw and saw the ball falling either left or right into one of the two boxes (the seesaw was manipulated from behind a curtain by a second experimenter). The experimenter did not follow the ball trajectory with her gaze but kept her eyes on the middle of the seesaw. After the ball fell, the seesaw was brought back into horizontal position. The experimenter then pushed the boxes to the edge of the table and asked the infant “Where is it?” Just like in familiarization, infants received two more prompts before ending the test trial. Unlike in familiarization, infants were given neutral feedback by being congratulated regardless of their choice, and the ball was *not* removed from the box. No infant tried to open the boxes after expressing their choice. A trial ended when infants chose a box or after the third prompt. Infants were then handed one of the two toys used in familiarization and encouraged to play with it while the experimenter set up the next trial by pulling the boxes backwards and placing a new ball in the middle of the seesaw. Each infant received four test trials.

### Design

The box in which the object was placed alternated across familiarization and test such that the toy in the last familiarization trial and the ball in the first test trial always ended up in opposite boxes (AB-ABBA). The side with which the AB-ABBA alternation started (left vs. right), the side of the boxes (orange right, blue left vs. orange left, blue right), and the experimenter’s position during the test question (to the left vs. to the right of the seesaw) were counterbalanced.

### Coding

We had two primary dependent measures: choice and correctness. Infants received a score of 1 for having made a choice if they unambiguously reached, grasped, or pointed to one of the two boxes, and 0 otherwise. Correctness was coded as 1 if they chose the box that was on the same side as the falling event, and as 0 otherwise. Infants’ responses were recorded by one researcher during the testing session, and double-coded from video by a second researcher who was blind to the ball location. Interrater reliability was very high (Cohen’s *κ* = .858); inconsistencies were solved by discussion. Based on piloting data, we preregistered a secondary measure and coded how often children pointed to the center of the seesaw when not choosing one of the two boxes.

### Exclusions

Based on preregistered criteria, we excluded four additional infants who did not make two consecutive correct choices in 8 familiarization trials. One additional infant was excluded due to experimenter error. We excluded trials in which infants did not follow the ball trajectory with their gaze based on video recordings (*n* = 2, out of 64 trials). One additional trial was excluded due to experimenter error.

### Data Analysis

Infants’ raw scores for each trial (0 or 1 for choice, 0 or 1 for correctness if infants made a choice) were supplemented by two additional individual scores: the proportion of choices across valid trials, and the proportion of correct responses across trials where a choice has been made. Since the balls that fell into the boxes throughout the test were not removed from the boxes, infants’ responses during later trials might be influenced by the fact that balls kept piling up in *both* boxes. Therefore, we also preregistered and ran a separate analysis for the first trial. All analyses were conducted in R 4.0.3 (R Core Team, 2021).

## RESULTS AND DISCUSSION

As expected, infants were able and motivated to solve the task. Most of them provided at least one response (87.5%, 14 out of 16 participants), and they did so in 72.1% of the trials (44 out of 61). When they made a choice, their responses were correct 83.3% of the time (*Mdn* = 100%, Wilcoxon signed rank, *V* = 82.5, *p* = .007, *r* = .655), well above the 50% chance level. Ten of the 16 infants performed at ceiling, never choosing the wrong box. On the first trial, of the 12 infants who chose a box, 11 were correct (binomial exact test, *p* = .006).

The purpose of Experiment 1 was twofold: (i) to make sure that infants can follow the trajectory of balls falling into boxes; and (ii) to get a quantitative baseline of this capacity when the entire setup consists of real objects. The results indicate that 19-month-olds can answer questions about displaced objects reliably and accurately. This benchmark allowed us to proceed to the main question of the study and investigate whether infants would do the same in a situation in which screen events appear to extend into the surrounding environment.

## EXPERIMENT 2: CROSSOVER

This experiment provided infants the same visual information about the location of falling balls as in Experiment 1, but now the animated balls fell from a cartoon seesaw on a TV screen, while the target locations were the same real boxes as in Experiment 1.

### Methods

Except for the details specified next, the methods were the same as in Experiment 1.

### Participants

The final sample consisted of 16 typically developing 19-month-olds (*M*_age_ = 19 months 7 days, *SD*_age_ = 13.9 days).

### Apparatus and Materials

We used an LCD TV screen (16:9, diagonal 110 cm) to play animations, in which a ball on the screen fell either to the left or the right. The same boxes used in Experiment 1 were placed under the screen to create the illusion that the ball lands into them (Figure 1, center).

### Stimuli

We transposed the events from Experiment 1 in a 2D-animated format, using *Adobe Animate CC 2018*: a red ball (more precisely, a red circle) falling off a seesaw to the left or to the right. The dimensions of the animated ball and seesaw matched those of the real objects. To give the illusion that the animated ball fell into the box, the boxes were placed under the screen based on ball trajectory. Black sheets extending from the boxes were used to cover the screen bezels to make the endpoint of the ball falling event ambiguous (see Figure 1).

### Procedure

#### Familiarization

The warmup phase was identical to Experiment 1: the experimenter dropped a (real) toy into one of the two boxes and asked the infant where the toy was.

#### Test

Test trials followed the same logic as those in Experiment 1. While behind the screen, the experimenter drew the infant’s attention to the red ball on the screen (“[Name,] look at the ball!”), which then rolled to the left or to the right of the seesaw. The experimenter then pushed the boxes toward the infant and asked them “Where is it?” The trial ended if the infant chose one of the two boxes or if they did not respond on the third prompt.

### Coding

Responses were recorded by one researcher during the testing session, and double-coded from video by a second researcher who was blind to the side on which the ball had fallen. Interrater reliability was substantial (Cohen’s *κ* = .761); inconsistencies were solved by discussion. As in Experiment 1, we also coded how often children pointed to the center of the screen as a secondary measure.

### Exclusions

We excluded four additional infants who did not make two correct choices in a row across eight familiarization trials and trials in which infants did not look at the falling event (*n* = 2). Two additional trials were excluded due to experimenter error.

## RESULTS AND DISCUSSION

Unlike in Experiment 1, only 50% of the infants chose a box at least once during test (8 out of 16 participants). Out of the 60 valid trials included in the final analysis, infants picked a box in 18 trials only (30%). Our secondary measure allowed us to rule out that infants were less motivated to provide an answer to the question in this version of the task: in 24 out of the remaining 42 trials (57%), infants pointed to the screen when asked where the ball was. When they did make a choice, infants chose the box that was on the same side of the falling event 45.8% of the time (*Mdn* = 0.5, Wilcoxon signed rank, *V* = 3.5, *p* = .71, *r* = .196). On the first trial, of the eight infants who chose a box, four were correct (binomial exact test, *p* = 1).

In the *Crossover* version of the falling ball experiment, infants behaved in a way that is inconsistent with the belief that animations are spatiotemporally continuous with reality. In contrast to Experiment 1, they were less likely to choose a box when asked where the ball was, and often preferred to point to the screen. When they did provide a response, however, they chose boxes at random instead of basing their answers on the side in which the ball was seen falling.

## EXPERIMENT 3: ANIMATION

It is possible that infants simply did not get the intended referent of the question “Where is the ball?” in Experiment 2 because they did not see the red animated circle as a potential candidate for “ball,” and that they pointed to the screen to request another animation. To rule out this alternative explanation, we added the two boxes to the animated world. If infants understand the question as we intended them to, they should now be able to point (again) to the correct location when asked about the whereabouts of the ball.

### Methods

Except for the details specified next, the methods were the same as in Experiment 1.

### Participants

The final sample consisted of 16 typically developing 19-month-olds (*M*_age_ = 19 months 3 days, *SD*_age_ = 12.8 days).

### Procedure

The procedure was the same as in Experiment 2 except for the boxes, which were also part of the animation during the test trials (Figure 1, right). The familiarization trials were identical to the ones in Experiments 1 and 2, but once infants passed the familiarization phase with the two plush toys, the cardboard boxes were removed from the table. Unlike in the first two experiments, the animated boxes were not brought closer to the infant after the test question was asked.

### Coding

Responses were recorded by one researcher during the testing session, and double-coded from video by a second researcher who was blind to the ball location. Interrater reliability was very high (Cohen’s *κ* = .804); inconsistencies were solved by discussion.

### Exclusions

We excluded five additional infants who did not provide two consecutive correct responses in eight familiarization trials. Three additional infants were tested but not included in the final sample: two infants because of experimenter error and one infant who did not look to the screen in any of the four trials. From the final sample, we excluded two trials: one in which the infant did not attend to the screen during the falling event and one due to experimenter error.

## RESULTS AND DISCUSSION

Comparable to the Experiment 1, 81.3% of infants gave at least one response (13 out of 16 participants). Out of the 62 valid trials included in the final analysis, infants chose a box in 30 trials (48.4%). As for accuracy, infants chose the box that was on the same side of the falling animated ball far from the 50% chance level: they pointed to the correct box in 93.6% of the trials in which they made a choice (*Mdn* = 1, Wilcoxon signed rank, *V* = 78, *p* < .001, *r* = .864). On the first trial, of the 11 infants who chose a box (out of 15; one participant’s first trial was excluded), 10 were correct (binomial exact test, *p* = .012).

While they made fewer choices overall compared to Experiment 1, infants overwhelmingly pointed to the box into which they saw the animated ball last fall on trials where they made a choice. This suggests that the random pattern of pointing in Experiment 2 was due neither to infants’ inability to link the animated red circle to the intended referent of “the ball” nor to other differences between Experiments 1 and 2 (e.g., the fact that the experimenter could not herself see the ball because she was standing behind the TV screen in Experiment 2).

## COMPARISONS ACROSS EXPERIMENTS 1–3

### Frequentist Analyses

#### Choices

**Figure 2.**
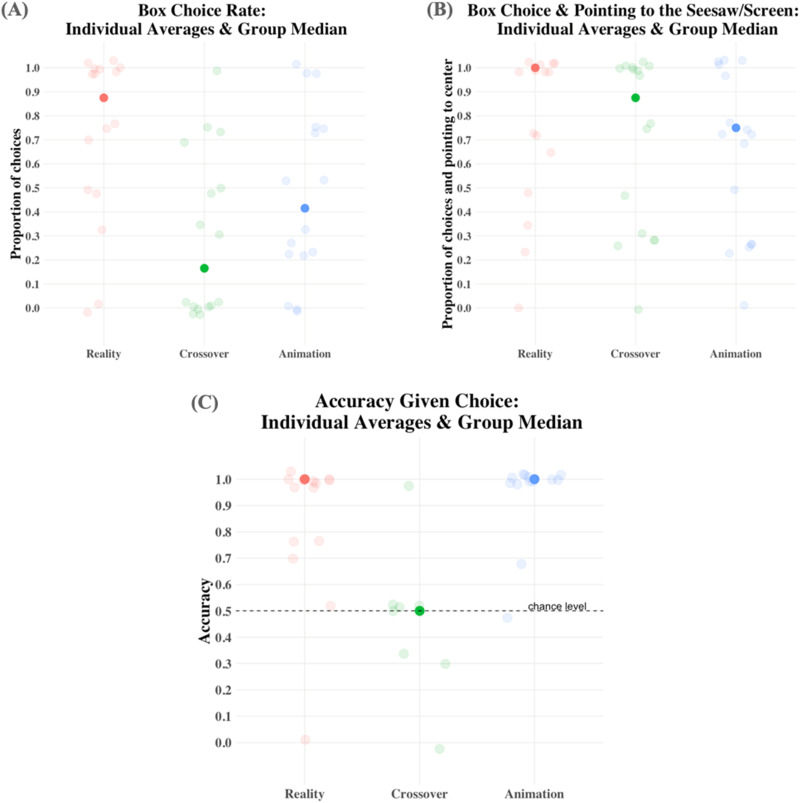
**Results of Experiments 1–3.** Transparent dots indicate individual proportions across the four trials; opaque dots represent group medians. (**A**) How often infants pointed to one of the two boxes in response to the test question. (**B**) How often infants pointed either to one of the two boxes or to the center of the seesaw/screen. (**C**) Proportion of correct responses in the trials in which infants chose one of the two boxes.

The experiment-wise box choice rates are displayed in Figure 2A. Nonparametric analyses revealed that the frequencies with which infants chose in the three experiments were unlikely to come from the same distribution, Kruskal-Wallis, χ^2^(2) = 9.361, *p* = .009. Planned pairwise comparisons with Holm’s correction reveal that it was the contrast between Experiments 1 and 2 that drove this difference (Dunn’s Test, *z* = 3.054, *p* = .007).

However, the frequency of responses to the question “*Where is the ball?*” did not differ across the three experiments. If we take into account how often infants pointed to the center of the display in front of them (our secondary measure), the difference between response rates disappears, Kruskal-Wallis, χ^2^(2) = 1.9, *p* = .386. Infants in Experiment 2 chose to point to the screen instead of the two boxes, even though they were made salient by the experimenter pushing them toward the infant before asking them where the ball was (Figure 2B). This strengthens the interpretation that they did not think the animated ball could have landed in the boxes below the screen.

#### Accuracy

Like choice rates, accuracy rates across the experiments (Figure 2C) were unlikely to come from the same distribution, Kruskal-Wallis, χ^2^(2) = 13.658, *p* = .001. This difference was driven by Experiment 2, where infants were at chance when choosing between the two boxes (Experiment 1 vs. 2, Dunn’s Test, *z* = 2.876, *p* = .008; Experiment 2 vs. 3, Dunn’s Test, *z* = 3.612, *p* < .001). When infants chose a box in Experiments 1 and 3, they chose it based on the falling event they had just seen. By contrast, in Experiment 2, they completely disregarded the animated falling event and picked one of the two boxes at random.

#### Bayesian Analysis

**Figure 3.**
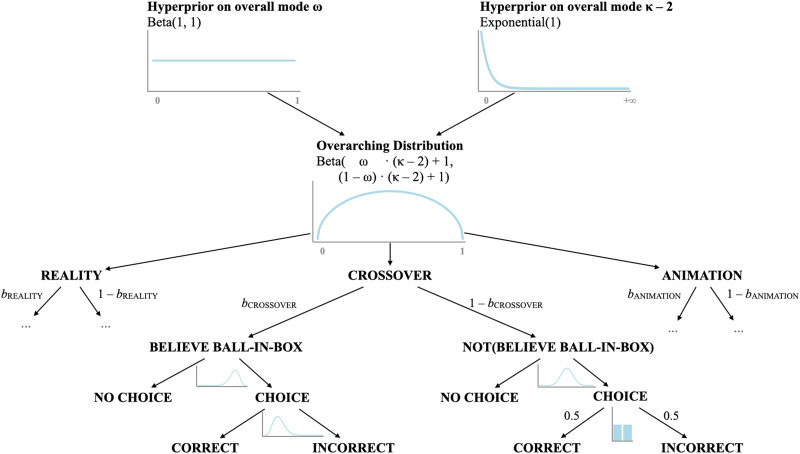
**Schematic representation of the data-generating process assumed to underlie infants’ choice and accuracy rates in Experiments 1–3.** Infants’ beliefs that the ball is in the box are generated from the same overarching distribution parameterized by ω and κ. In each of the three experiments and in each trial, infants can either choose a box or not and, if they do, they can choose it correctly or not. From the observed behavior, the tree can be inverted via Bayes’ rule to obtain infants’ beliefs in each of the three experiments.

To model both choice and accuracy rates, we built a hierarchical Bayesian multinomial mixture model in STAN (Carpenter et al., 2017; Kruschke, 2015, p. 759; McElreath, 2020), which considers the two independent measures at once (Figure 3). Using infants’ responses (*no choice*, *correct choice*, or *incorrect choice*), the model allows us to infer both (i) whether infants believe falling balls end up in boxes, and (ii) whether their beliefs differed across experiments. We use *b*_EXPERIMENT_ (ranging from 0 to 1, one for each Experiment) to denote infants’ beliefs about ball location in each experiment and place a weak prior on the three *b*-values, centered on 0.5 and skeptical of extreme values. We make three assumptions as to how beliefs and responses are linked. First, we assume that infants are more likely to make a choice *and* to choose correctly if they believe that the ball is in one of the two boxes (indicated by the mildly skewed priors on the left side of the tree). Second, we assume that infants are equally likely to refrain or to choose a box (at random) when they do not think that the ball is in either of the two boxes (as shown by the balanced priors on the right side of the tree). Third, to avoid overfitting the differences among experiments, we assume that the *b*-parameters are sampled from the same underlying beta-distribution (parameterized by ω and *κ*). The scripts to replicate the analyses can be found on the Open Science Framework (OSF) project page (https://osf.io/s83qn).

Having constructed the data-generating model (from infants’ beliefs to their responses), we use Bayes’ rule to invert it to infer infants’ beliefs from their responses. In the extreme case, if infants always choose *and* choose correctly, they probably believe that the ball is in the box (left side of the tree). On the other hand, if infants make a choice only half of the time, and are at chance when choosing, they probably do not think that the ball is in the box (right side of the tree). Thus, large *b*-values (closer to 1) would indicate that infants believe there is a ball in the box into which they last saw it fall; conversely, small *b*-values (closer to 0) would indicate that infants do not entertain this belief.

**Figure 4.**
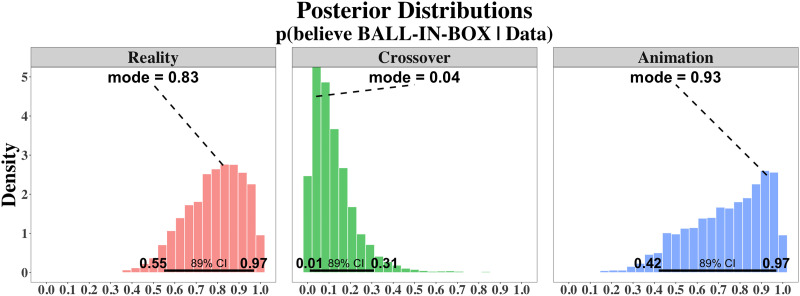
**Posterior distributions for the *b*-parameter in each of the three experiments.** Bold horizontal lines above the *x*-axis give the 89% credible interval of the distributions.

The posteriors on the overarching parameters *b*_EXPERIMENT_ (one for each experiment) are displayed in Figure 4. For Experiment 1, *b*_Reality_ peaks close to 1 (mode = 0.83, 89% credible interval = [0.55, 0.97]), suggesting that infants relied on the previous ball falling event when answering the test question. Similarly, *b*_Animation_ also peaks toward the right end of the [0, 1] interval, but the estimate is noisier because infants made fewer choices than in Experiment 1 (mode = 0.93, 89% credible interval: [0.42, 0.97]). By contrast, *b*_Crossover_ shows the opposite trend toward 0, indicating that infants did not think that the animated ball ended up in real boxes (mode = 0.04, 89% credible interval: [0.01, 0.31]).

## DISCUSSION

The results obtained in Experiments 1 to 3 allow us to rule out two of the three hypotheses we have started with. On the one hand, infants did not behave as a naïve-realism account would predict (Hypothesis 2). When asked where the ball was in Experiment 2, they either pointed to the screen or chose one of the boxes at chance, indicating that they did not expect animated balls falling on-screen to end up in boxes below the screen. However, this was not because the animation itself was too impoverished for them to link the red circle on the screen to the noun phrase *the ball* (Hypothesis 1). Otherwise, they would have failed in Experiment 3, where everything was on the screen.

However, it remains an open question whether infants have just learned that screens are *spatially* disconnected from their surroundings, while still believing that the events depicted on the screen are happening *here and now*, just like in an aquarium (Hypothesis 3). If this is the case, infants should not accept that an event displayed on one screen can move to a different screen—unlike adults, who can start watching a movie in the theater and end it on their laptops at home without losing track of narrative continuity. This potential explanation was tested in Experiment 4.

## EXPERIMENT 4: AQUARIUM

Experiment 4 asked how infants would identify the protagonist of an animation when they get potentially conflicting information about its location. We showed infants two animations on two different screens, placed side-by-side on a table. Each animation consisted of an animal (a bear and a rabbit, respectively) leaving its house and entering back in. The houses were identical, but the animation backgrounds were different. After making sure that infants learned which animal lived on which screen, we surreptitiously swapped the two backgrounds and asked infants about the animals’ location again. Do infants individuate the protagonists by their physical locations (the house in the screen on which the animation was presented) or by their virtual locations (the house in the animation scene of which the protagonist was a part of)? If they opt for the virtual location, the aquarium hypothesis can be ruled out: screens are not merely spatially bounded physical containers for infants.

### Methods

#### Participants

The final sample consisted of 32 typically developing 19-month-olds (*M*_age_ = 19 months 17 days, *SD*_age_ = 7.63 days).

#### Stimuli

We created two 15-s animations featuring two protagonists, a rabbit and a bear (Figure 4). In each animation, the protagonist came out of its house, walked around, fetched a piece of fruit, then went back inside. Crucially, the backgrounds of the two animated worlds were chosen to contrast as much as possible, but the animals’ houses were identical. In addition, we also prepared two 5-s animations that showed the two animals exiting the house and entering back in (see *Familiarization*).

#### Design

The experiment consisted of two between-subject conditions^1^ and a single trial. The two conditions differed in whether the animation backgrounds were swapped (Swap Condition) or not swapped (No-Swap Condition) between monitors from familiarization to test. We used a single trial because subsequent trials would have been tainted by evidence (from the first trial) that animations can move from one screen to another.

#### Apparatus

**Figure 5.**
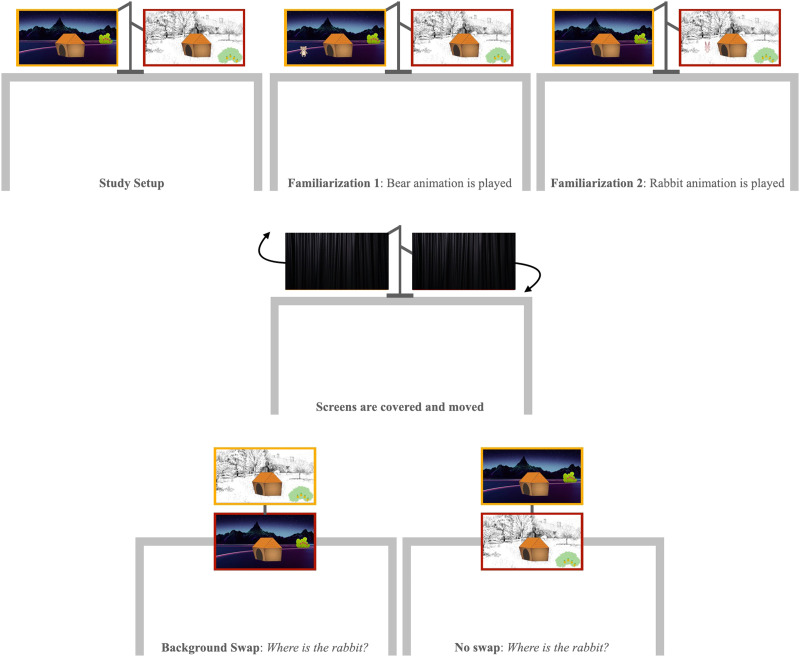
Schematic representation of Experiment 4.

We used two LCD monitors (16:9, diagonal 61 cm) to play the two animations. The monitors were held by a VESA dual mount arm, suspended above a table (Figure 5). Because we wanted to help infants with keeping track of the physical monitors, we placed differently colored tapes on the bezels of the two monitors. We glued two curtains to the monitors (one for each monitor), to be able to cover them between familiarization and test, so that infants could track the movement of both screens individually.

### Procedure

#### Familiarization

Infants were seated on their caregivers’ laps on a chair, approximately 40 cm from the table on which the monitors were placed. Caregivers were instructed at the beginning of the session to close their eyes during the test phase. The experimenter moved next to the infant and (in infant-directed intonation) drew their attention to one of the two screens (“Oh, look, here is the bear’s house. Let’s see what’s going to happen!”). Then corresponding animation started playing, and the experimenter narrated the events unfolding on the screen (e.g., “Wow, look a bear! The bear comes out of the house. And look, he’s walking! Oh, and now the bear is collecting a raspberry and then he’s going back! He’s entering the house again!”). After the animation was over, the experimenter went behind the screen, and asked infants where the animal was (e.g., “Where is the bear? Can you show me?”). If infants did not answer within 3 s, the experimenter repeated the question once or twice. If infants did not answer or answered incorrectly, they were shown a 5-s clip showing the bear (rabbit) coming out of the house and going back in. The question(s) were then repeated, and the short clip was shown once more if infants did not respond. If and when they responded correctly (by pointing to the screen on which they had just seen the animation), the experimenter congratulated them, and repeated the same process with the second screen and animation.

#### Pretest

After passing the second familiarization question, infants were asked about the first animal again, because we wanted to make sure that they had stored the locations of *both* animals. If they failed to answer the question correctly, the familiarization phase was repeated once. If infants answered correctly, they were congratulated, and the test phase started.

#### Test

The experimenter drew the curtains over the two monitors and brought them from horizontal to vertical alignment (Figure 5, bottom row). This manipulation was meant to eliminate side and perseveration biases. During the rearrangement, the two monitors remained visible at all times, such that infants could track the individual screens through space. In the Swap Condition, we surreptitiously swapped the two backgrounds while the screens were covered; in the No-Swap Condition, nothing else changed. Once the monitors were vertically aligned, the experimenter unveiled them by pulling the curtain backwards, moved next to the child, and asked about one of the two animals’ whereabouts: “Look what’s happening! Let’s find the animals! Where is the bear (rabbit)?” If the infant did not provide a response within 3 s, the experimenter asked two more questions (“Can you show me the bear (rabbit)? In which house is the bear (rabbit)?”). Once infants pointed to one of the two screens, they were asked the same question about the remaining animal. The responses to this second question were not analyzed (as they were not independent from the responses to the first one), but we included it to make sure that infants were answering the location questions consistently (if they think the bear is on screen A, they should also think that the rabbit is on screen B). Otherwise, it would be unclear whether their pointing was related to the test question at all (e.g., it could mean “I want to see that animation again”). Thus, infants with inconsistent answers were excluded.

Which animation went on which screen (left vs. right), which animation was played first (bear vs. rabbit), the content of the test question (*Where is the bear?* vs. *Where is the rabbit?*), and the experimenter’s position during the test question (right vs. left) were counterbalanced across participants in both conditions.

### Coding

Responses (upper vs. lower screen choice) were recorded by one researcher during the testing session, and double-coded from video by a second researcher who was blind to the animals’ locations. Interrater reliability was very high (Cohen’s *κ* = .812); inconsistencies were solved by discussion.

### Exclusions

We had two main criteria for inclusion in the final sample. First, infants had to provide *three* consecutive alternating answers during familiarization to make sure that they stored both animals’ locations in memory before we covered the screens. Second, infants had to provide contrastive answers at test. If they pointed to one screen in response to the *bear*-question, they had to point to the other screen in response to the *rabbit*-question. Even though we only analyzed the first answer, we sought to make sure that they answer the location question. If they point to the same screen when asked about the two different animals, their pointing might express a preference for one of the two animations instead of reflecting their beliefs about the animals’ locations. In addition, we used a single trial to avoid the possibility that infants learn across trials that these two particular screens swap their contents when covered.

Despite almost no drop-out during piloting, we had to exclude 28 infants from the final sample based on these preregistered criteria, because they did not pass familiarization (*n* = 14; 6 in the *Swap* condition, 8 in the *No-Swap* condition), did not provide a contrastive answer at test (*n* = 10), or did not answer at all (*n* = 4). In addition, we excluded 13 infants due to experimenter and technical errors (*n* = 8), fussiness (*n* = 4), and parental interference (*n* = 1).

## RESULTS

Before proceeding to data analysis, infants’ up-down scores for each trial were recoded to represent infants’ strategy at test. They received a score of 1 (*stay*) if they pointed to the *same physical screen* they pointed to at familiarization and a score of 0 (*switch*) if they pointed to *the other screen* than the one chosen in familiarization. By this coding scheme and under the assumption that the virtual location is the correct answer, 1 (*stay*) is the correct response in the No-Swap condition, while 0 (*switch*) is the correct response in the Swap condition.

### Frequentist

In the Swap Condition, 12 out of 16 babies pointed to a *different* screen from the one they pointed to during familiarization—they went for the virtual location as opposed to the physical one. In the No-Swap Condition, where there were no conflicting location cues, 13 out of 16 babies pointed to the *same* screen as during familiarization. The observed effect of conditions was unlikely under the null hypothesis (Fisher’s exact test, *p* = .004). When we added the responses of infants who were excluded because they had not provided a contrastive answer to the control question, the effect of condition does not change: 14 of 20 participants chose the *different* screen in the Swap Condition and 17 of 21 participants chose the *same* screen in the No-Swap Condition (Fisher’s exact test, *p* = .002).

### Bayesian Analysis

**Figure 6.**
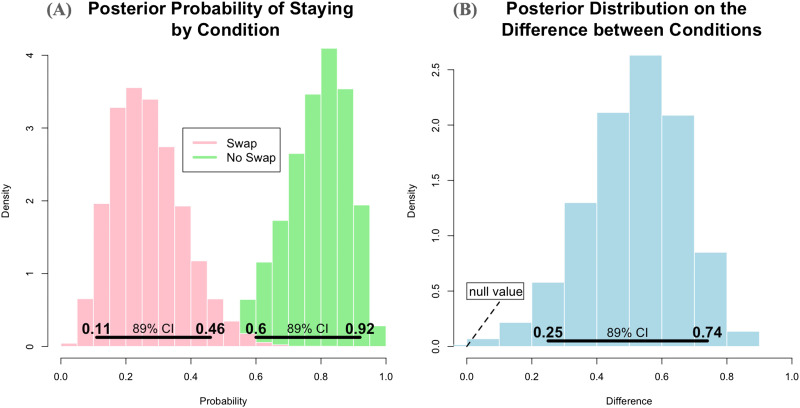
**Results of Experiment 4.** (A) Estimated probabilities of the two pstay distributions. In the No Swap condition, infants choose the same screen as in familiarization (as expected). By contrast, in the Swap condition, the probability to choose the same screen is low, indicating that they individuate the animated animals by background. (B) Posterior difference between the distributions in (A). Bold horizontal lines above the x-axis give the 89% credible interval of the distributions.

To obtain the probability of staying in each condition separately, as well as the difference between the two conditions, we use a Bayesian logistic regression model. The details of the model and the scripts to replicate the analyses can be found on the OSF project page (https://osf.io/s83qn). The posterior distributions of the probability of *staying* in the Swap vs. No-Swap Condition are shown in Figure 6A. In the No-Swap Condition, the posterior mean for this parameter was .78 (89% credible interval: [0.6, 0.92]), while in the Swap Condition, it was .27 (89% credible interval: [0.11, 0.46]). Figure 6B depicts the histogram of differences between conditions as estimated by the model. The posterior of differences indicates that infants’ responses are influenced by whether the backgrounds are swapped or not (89% credible interval excludes 0 as a plausible value).

## DISCUSSION

The results of Experiment 4 rule out a potential explanation (Hypothesis 3) for the results in Experiments 1 to 3. According to this explanation, infants rejected the apparent screen-reality crossover in Experiment 2 because for them screens are containers with rigid boundaries that do not allow objects to pass through. This account predicts that infants should identify animated characters based on the screen on which they are presented. However, infants linked the two protagonists to their virtual environments, not physical location, when the two possible locations were pitted against each other.

## GENERAL DISCUSSION

Investigating how infants interpret animated stimuli is relevant for both theoretical and methodological reasons. One prominent way in which humans communicate is through the use of symbols for representing entities they want to communicate about. Symbols and the actions performed on them, are used to create a physical scene through which events, relations, and properties of distal objects are depicted (Clark, 2016). Beyond animations, we find the same setup in graphs, assembly instructions, joint pretend play—representations where the visual and conceptual systems of the interlocutor are recruited for interpretation. The capacity to set up these links is central to gathering information about distal states of affairs from proximal sources, enabling us to widen the range of things we can learn about without first-hand experience. Thus, the ability to grasp and exploit representations lies at the intersection of communication and learning, and therefore understanding how it develops can inform debates on both of these topics.

In addition, representations are especially relevant for developmental methodology because they are pervasively used to elicit infants’ and children’s inferences (e.g., animations, puppet shows, games). Moreover, experimental setups involving TV-reality crossovers are used in developmental research under the assumption that infants are naïve realists. In Lucca et al. (2018), for instance, 13- and 17-month-old infants saw on a screen two people dropping objects, and were encouraged to go to one of the trays on the floor (as a measure of who they preferred between the two on-screen agents), where they would find the object dropped on-screen. Did infants really believe that these are the same objects they saw on the screen? At the heart of these methodologies lies the tacit assumption that infants take these stimuli at face value. The experiments reported here brought this assumption to the surface and provided a straightforward way for testing it. Our results do not invalidate studies that used screen-reality crossovers in their designs (for one, infants in the study mentioned above were younger than those in our sample). Rather, they do highlight the fact that assumptions that underlie methodological decisions should be empirically tested.

We outlined four hypotheses in the Introduction (Table 1), three of which are incompatible with the results in Experiments 1–4. The *full opacity* account (Hypothesis 1) predicts that infants would not be able to understand animated falling events as such and would thus fail in both Experiments 2 and 3. Infants had no problems, however, with tracking the trajectory of animated balls within the confines of the screen (Experiment 3). If the *naïve realism* account (Hypothesis 2) were true, infants would represent animation and reality as a spatial continuum, and we should see the same pattern of results across the first three experiments. This is not what we found. When infants were faced with an animation that appeared to continue beyond the screen, they were not fooled into thinking that the boundary could in fact be crossed (Experiment 2). When asked where the ball was, infants either ignored the boxes pushed toward them and pointed to the screen or picked one of the boxes at random. Finally, while the *aquarium* account (Hypothesis 3) can accommodate the results from the first three experiments, it cannot explain why infants identified animated characters by the background of the animation, as opposed to their physical location in Experiment 4.

**Table 1.** An overview of the predictions made by the four different accounts for Experiments 1–4 and the observed results.

	**Reality** 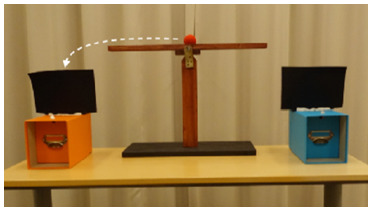		**Crossover** 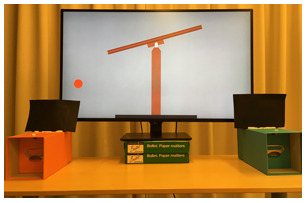		**Animation** 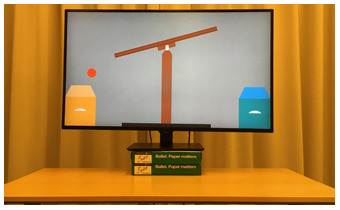		**Aquarium** 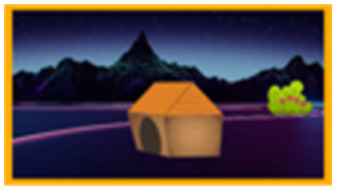	
	**predicted**	**observed**	**predicted**	**observed**	**predicted**	**observed**	**predicted**	**observed**
**1. Full opacity**	✓	✓	×	×	×	✓	×	✓
**3. Naïve realism**	✓		✓		✓		×	
**3. Aquarium**	✓		×		✓		×	
**4. Representation**	✓		×		✓		✓	

*Note*. Checkmarks represent above-chance performance (Experiments 1–3) or a difference between conditions (Experiment 4); crosses represent chance levels (Experiments 1–3) or no difference between conditions (Experiment 4). The observed results support Hypothesis 4.

While the results reported here do not provide direct evidence that infants at this age interpret animations as representations of (real or fictional) states of affairs, the responses that we recorded are compatible with an early concept of representation (Hypothesis 4). To recapitulate, we do not think representations are defined by reference to the world, but by the *stand-for* relation between a physical symbol—unitary pixel constellation on the screen, marks on paper, props—and a conceptually defined entity about which information is conveyed. This formulation of external representations renders the format of representation (*x* stands for *y*) independent of the content (*y* may or may not be a particular thing in the world). In a typical pretend play scenario, for instance, when 2-year-olds pretend that a wooden block is a carrot (Harris & Kavanaugh, 1993), they do not take the block to stand for a particular carrot in the world. Rather, they use their conceptual system to generate a new carrot token for the occasion. By contrast, in the tasks used by DeLoache and colleagues (reviewed in DeLoache, 2004), relying on this mechanism will not do, as the object symbol represents another particular object in the world, not merely a conceptually defined entity.

Note that the *dual representation* explanation (DeLoache, 2004) cannot account for the contrast between infants’ behavior in our experiments (or their early proficiency with pretend play) and their failures in the tasks used by DeLoache and colleagues. The dual representation account attributes the failures to a deficiency in the representation of the object symbol (both an object and a stand-in for something else). However, animations and pretend play build on the same duality (both a 2D circle and a stand-in for a ball; both a block and a stand-in for a carrot), yet infants and young toddlers respond appropriately in these scenarios. We speculate that it is the nature of the referent that underlies this difference instead. When the referent is not a particular object, infants set up the appropriate stand-for relation between a physical symbol and a conceptually defined entity. When the referent is a particular object, they struggle with the tasks because they fail to make the additional link from the conceptually defined entity to the particular object they need to retrieve.

If infants were able to set up stand-for relations between a visual object and a conceptually defined entity, their responses in our experiments would be naturally accounted for. In Experiments 2 and 3, infants linked the definite noun phrase *the ball* to the red circle on the screen (without explicit instruction) and were able to answer questions about *the ball* by tracking the trajectory of the red circle. Since animated objects do not exit screens, infants’ responses diverged from the crossover to the fully animated setup. However, this was not merely due to the physical boundary of the screen, or else they would have rejected the possibility that the bear and rabbit could have swapped locations in Experiment 4. But since animated bears and rabbits are not actual agents, infants did not individuate them based on physical location, but tracked the cues to the symbols presented in familiarization instead (i.e., the animated backgrounds).

It goes without saying that experience with animations and screens, with which the infants in our sample had extensive contact prior to their lab visit, is necessary in order to understand (i) that this particular class of stimuli is (potentially) representational; and (ii) how the representational medium works (screens, in this case). There is no reason to expect that sampling from a population of infants who have no experience with animations would have produced the same results as the ones presented here. Our participants’ prior experience with animations was a precondition that allowed us to test whether infants interpret certain classes of stimuli as coherent representations of entities belonging to familiar classes (balls, animals).

Finally, we would like to highlight two questions that the studies reported here do not answer. The first open question concerns the role of the experimenter who interacted with the infants throughout the test session. While the experimenter did not explicitly link symbols (i.e., the red circle) and referents (i.e., the ball) in Experiments 1–3, she did scaffold infants’ interpretations by providing labels (“ball,” “rabbit,” “bear”) that could be mapped onto the visual objects on the screen. It is therefore unclear whether infants would interpret animations in the same way if left to their own devices, and it remains an open question what scaffolding elements infants need to interpret animations as they did in our studies.

The second open question concerns the interpretation infants would give to other classes of stimuli, such as videos, which were not tested in the current studies. Given our data, we cannot exclude the possibility that a setup like the one in Experiment 2 with video recordings instead of animations might fool infants into accepting the screen-reality crossover. However, a direct comparison with other classes of stimuli would go beyond the scope of the current project. Our data should be taken as a proof-of-concept that the interpretation of certain stimuli (i.e., animations) is compatible with an early understanding of representations, not as evidence that infants have mastered the full ontology of their environment. Even if infants were to reject a video-reality crossover in a setup such as the one in Experiment 2, virtual reality or realistic holograms would most likely lead them into error. Our goal, however, was not to fool infants, but to investigate their behavior in response to stimuli that do *not* fool them.

Taken together, the data we obtained point to several conclusions. First, the world of infants is not a continuous spatiotemporal hodgepodge, as they do not confuse represented events with the immediate environment of animations. By 19 months, they have figured out that what happens on-screen stays on-screen, and they can answer questions about the location of objects appropriately based on this knowledge. Second, they have also figured out that animations are independent from the physical location they are presented at. In other words, they dissociate medium and content, just like adults do. We take this as preliminary evidence for the claim that infants of this age and from an industrialized population might already interpret animated objects and events as representations.

## ACKNOWLEDGMENTS

We thank Iulia Savos, Bálint Varga, and Dori Mészégető for data collection and coding, Ádám Koblinger for Bayesian guidance, and Laura Schlingloff, Gabor Brody, Denis Tatone, and Dan Sperber for discussions.

## FUNDING INFORMATION

GC, European Research Council (https://dx.doi.org/10.13039/501100000781), Award ID: 742231.

## AUTHOR CONTRIBUTIONS

BR: Conceptualization: Equal; Formal analysis: Lead; Methodology: Equal; Visualization: Lead; Writing - Original Draft: Lead; Writing - Review & Editing: Equal. GC: Conceptualization: Equal; Formal analysis: Supporting; Methodology: Equal; Visualization: Supporting; Writing - Original Draft: Supporting; Writing - Review & Editing: Equal; Funding Sources.

## Note

^1^ A slightly different version of the same study was preregistered on the OSF because we initially thought that running the Swap Condition only would suffice to test the aquarium hypothesis (*n* = 32). After the preregistration, we realized that the results from the experimental condition (Swap Condition) would not be interpretable without a control condition, so we decided to split the sample into two equal (*n* = 16) groups.

## Supporting Materials

https://osf.io/bwu9p

https://osf.io/juerf/

https://osf.io/gj5ys/

https://osf.io/s83qn
